# Case Report: Severe Gonadal Dysgenesis Causing 46,XY Disorder of Sex Development Due to a Novel *NR5A1* Variant

**DOI:** 10.3389/fgene.2022.885589

**Published:** 2022-07-05

**Authors:** Kheloud M. Alhamoudi, Balgees Alghamdi, Abeer Aljomaiah, Meshael Alswailem, Hindi Al-Hindi, Ali S. Alzahrani

**Affiliations:** ^1^ Department of Molecular Oncology, King Faisal Specialist Hospital and Research Centre, Riyadh, Saudi Arabia; ^2^ Department of Medicine, King Faisal Specialist Hospital and Research Centre, Riyadh, Saudi Arabia; ^3^ Department of Pathology and Laboratory Medicine, King Faisal Specialist Hospital and Research Centre, Riyadh, Saudi Arabia

**Keywords:** NR5A1 mutation and gonadal dysgenesis, DSD, disorders of sex development, ambiguous genitalia, gonadal dysgenesis, NR5A1

## Abstract

Mutations in the nuclear receptor subfamily 5 group A member 1 (*NR5A1*) are the underlying cause of 10–20% of 46,XY disorders of sex development (DSDs). We describe a young girl with 46,XY DSD due to a unique novel mutation of the *NR5A1* gene. An 11-year-old subject, raised as a female, was noticed to have clitromegly. She looked otherwise normal. However, her evaluation revealed a 46,XY karyotype, moderate clitromegly but otherwise normal female external genitalia, undescended atrophied testes, rudimentary uterus, no ovaries, and lack of breast development. Serum testosterone and estradiol were low, and gonadotropins were elevated. Adrenocortical function was normal. DNA was isolated from the peripheral leucocytes and used for whole exome sequencing. The results were confirmed by Sanger sequencing. We identified a novel mutation in *NR5A1* changing the second nucleotide of the translation initiation codon (ATG>ACG) and resulting in a change of the first amino acid, methionine to threonine (p.Met1The). This led to severe gonadal dysgenesis with deficiency of testosterone and anti-Müllerian hormone (AMH) secretion. Lack of the former led to the development of female external genitalia, and lack of the latter allowed the Müllerian duct to develop into the uterus and the upper vagina. The patient has a female gender identity. Bilateral orchidectomy was performed and showed severely atrophic testes. Estrogen/progesterone therapy was initiated with excellent breast development and normal cyclical menses. In summary, we describe a severely affected case of 46,XY DSD due to a novel *NR5A1* mutation involving the initiation codon that fully explains the clinical phenotype in this subject.

## Introduction

Disorders of sex development (DSDs) are a group of congenital conditions characterized by impaired development of chromosomal, gonadal, and/or phenotypic sex (Ono and Harley, 2013). Depending on the etiology of the disease, DSDs have been classified into several categories including sex chromosome DSDs, 46,XX DSDs, and 46,XY DSDs (Witchel, 2018). The 46,XY DSDs are characterized by ambiguous external genitalia with a 46,XY karyotype (García-Acero et al., 2020). The 46,XY DSD patients, however, are known to be phenotypically and etiologically heterogeneous and may develop variable genital manifestations.

The ongoing advances in the genomics applications have been instrumental in revealing the etiology of several DSDs (Baxter et al., 2015; Audi et al., 2018; Witchel, 2018). Numerous genes have been associated with 46,XY DSD such as ARX, ATRX, CBX2, DHH, DMRT2, FGFR2, GATA4, MAP3K1, NR0B1, NR5A1, SOX9, SRY, TSPYL1, WNT4, WT1, WWOX, ZFPM2, AKR1C2, AKR1C4, AMH, CYB5A, CYP11A1, CYP17A1, HSD17B3, HSD3B2, LHCGR, POR, SRD5A2, STAR, AMHR2, and androgen receptor (AR) (Audi et al., 2018). In the last few years, Nuclear receptor subfamily 5 group A member 1 (*NR5A*1) mutations have been detected in about 10–20% of 46,XY DSD cases as the major causes of gonadal dysgenesis in males and ovarian insufficiency in genetic females (Ferraz-de-Souza et al., 2011; Voican et al., 2013; Suntharalingham et al., 2015).

*NR5A*1 (OMIM: 184757), also abbreviated as steroidogenic factor 1 (*SF*1) or adrenal 4-binding protein (*Ad4BP*), is a member of the nuclear receptor family (Morohashi and Omura, 1996). The *NR5A*1 gene resides on chromosome 9q33.3 and contains seven exons: one nontranslated exon (exon 1) and six other coding exons (exons 2–7) (Hoivik et al., 2010). Structurally, the NR5A1 protein consists of a DNA-binding domain (DBD), a ligand-binding domain (LBD), two functional activation domains (AF-1 and AF-2), and a hinge region. It has been demonstrated that NR5A1 is highly expressed in Sertoli and Leydig cells of the fetal and adult gonads as well as the adrenal cortex, placenta, ovary, testis, hypothalamus, and anterior pituitary (Luo et al., 1994; Parker et al., 2002; Zhao et al., 2007; Lin and Achermann, 2008; Ferraz-de-Souza et al., 2011).

To date, more than 150 disease-causing mutations in the *NR5A*1 gene associated with 46,XY disorders have been reported in the Human Gene Mutation Database (HGMD) database (Lin et al., 2007; Philibert et al., 2007; Köhler et al., 2008; Soardi et al., 2010; Allali et al., 2011; Tantawy et al., 2014). Here, we report a 46,XY patient with a female external genitalia phenotype, rudimentary uterus, primary amenorrhea, lack of secondary sexual characteristics, and bilateral undescended inguinal testes. We identified a novel heterozygous missense *NR5A1* variant (c.2T>C, p.Met1Thr) that explains her phenotype and adds to the spectrum of *NR5A1* mutations that cause gonadal dysgenesis and DSDs.

## Patient and Methods

### Human Subject and Ethical Approval

The proband underwent a comprehensive clinical, biochemical and radiological evaluation by an experienced endocrinologist and genetic evaluation and counseling by a geneticist. Ethical approval of this study was obtained from King Faisal Specialist Hospital and Research Centre Institutional Review Board (RAC number 2130-012). An informed consent was obtained from the patient and her parents.

### Genomic DNA Extraction and Screening of Candidate Genes

Genomic DNA was extracted from peripheral leucocytes using a commercial DNA extraction kit (QIAamp Blood Midi Kit, Qiagen, Hilden, Germany) according to the manufacturer’s instructions. Initial genetic testing was directed to the 5-alpha reductase (*SRD5A2*) and AR using Sanger sequencing of all exons and exon–intron boundaries according to previously reported methods (Alswailem et al., 2021). This showed no potential variants in any of these two genes.

### Whole Exome Sequencing

Whole exome sequencing (WES) was performed on the DNA of the affected proband using the Illumina HiSeq 2500 platform to capture regions of interest from the fragmented DNA library. A minimum coverage of 30× of 95% of the target regions was performed, and the sequence data from the proband was mapped to the human genome build UCSC hg19 reference sequence. The quality and coverage assessment for targeted coding exons of the protein-coding genes were ascertained.

### Bioinformatics Filtration Step

Primary filtering was performed using the standard method, including filtering out low-quality reads and potential artifacts. All phenotype-driven genes reported in the Human Phenotype Ontology, the HGMD, the 1000 Genomes database, the NHLBI GO Exome Sequencing Project, the OMIM and PubMed databases were considered. Briefly, copy number variation (CNV) calling was used. Following this, all disease-causing variants reported in the HGMD (Stenson et al., 2014), in ClinVar (Landrum et al., 2016), and in CentoMD in addition to all variants with minor allele frequency below 1% in the genomAD and the Exome Aggregation Consortium (ExAC) database (Lek et al., 2016) were given priority. Variant filtration steps also focused on coding exons and flanking ±20 intronic bases. All pertinent inheritance patterns were considered. The family history and patient clinical information provided were used to evaluate the variants that were eventually identified in genes associated with 46,XY DSD. Variants that were filtered after WES were characterized with respect to their pathogenicity and causality using the published American Collage of Medical Genetics and Genomics guidelines (Richards et al., 2015). All variants related to the phenotype of the patient, except for benign or likely benign variants, were reported. CNVs of unknown significance were not reported.

### Mutation Confirmation and Sequencing Analysis

Sanger sequencing was carried out to validate the identified variant. The identified missense mutation in the *NR5A1* gene (NM_004959.5, c.2T > C) was validated using primers: F: 5′-CGA​TCT​TGC​AGC​TCT​GGC-3′ and R: 5′-TCT​CAG​ACA​AAC​GAA​TCC​CAA-3′. A standard PCR using the platinum II Hot-Start Green PCR Master Mix was performed following the manufacturer’s instruction and an annealing temperature of 55°C. Direct sequencing reaction was performed using BigDye Terminator version 3.1, cycle sequencing reaction kit and an ABI PRISM 3730XL genetic analyzer (Applied Biosystems).

## Results

### Clinical Description

An 11-year-old person, raised as a girl, born to non-consanguineous parents, presented to our clinic after her mother noticed atypical genital appearance with large clitoris. The patient was born at full term *via* a normal vaginal delivery, with normal pregnancy history. She looked a normal baby girl and was not noticed to have any physical abnormality at birth. During childhood, she was raised as a normal girl with no history of abnormal behavior or significant medical events. She was the first child of five siblings younger than her. None of them was reported to have any genital or somatic abnormality. She had no family history of infertility, amenorrhea, or abnormal development of external genitalia.

At the time of presentation at 11 years of age, physical examination revealed no dysmorphic features. Vital signs were within normal limits with weight of 46 kg on the 50th centile, height of 135 cm on the 45th percentile, and BMI of 25.2. Her blood pressure was normal at 105/65 mm Hg. Genitalia examination revealed mild clitromegaly with one orifice for the urethral meatus and another inferior separate vaginal meatus, partially fused labioscrotal fold, normal hymen, and no masses in labioscrotal folds. There was a firm mass of about 2-cm length in the right inguinal canal. Breast examination showed Tanner stage I. Hair distribution was normal without facial, chest, or abdominal hair but normal early pubic hair (Tanner II). The rest of her physical examination was normal.

Laboratory workup showed creatinine 51 μmol/L (46–96), Na 142 mmol/L (135–145), K 4.2 (3.5–5.0), CO_2_ 24 mmol/L (22–31), follicle-stimulating hormone (FSH) 63 u/L (1.5–12.4), luteinizing hormone (LH) 31 u/L (1.7–8.6), testosterone 3.55 nmol/L (0.2–2.9), estradiol 36 pmol/L (28–156), progesterone 0.4 nmol/L (follicular phase levels, 0.6–4.7), 17α-hydroxyprogesterone 0.6 nmol/L (normal <3.0), dihydrotestosterone 87 pg/ml (normal <200), DHEAS 2.48 μmol/L (1.8–8.3), morning ACTH 38 ng/L (5–60), morning cortisol 208 nmol/L (166–507), and post-ACTH stimulation 580 nmol/L. The human chorionic gonadotropin stimulation test showed an increase in testosterone level from 3.5 to 7.5 nmol/L. Pelvis ultrasound examination showed vaginal pouch in the pelvis; however, the uterus was not visualized. There was an oval-shaped isoechoic structure noted in the right inguinal region, measuring 1.99 × 1.09 cm and shows inner vascularity. Pelvic magnetic resonance imaging showed hypoplastic small uterus measuring about 2.4 × 0.7 × 0.2 cm in craniocaudal and transverse diameter with small, thin endometrial stripe. The ovaries, seminal vesicles, and prostate could not be visualized. The cytogenetics results revealed that the patient had a 46,XY karyotype. Psychosocial evaluation repeatedly confirmed that she has a female gender identity.

**FIGURE 1:**
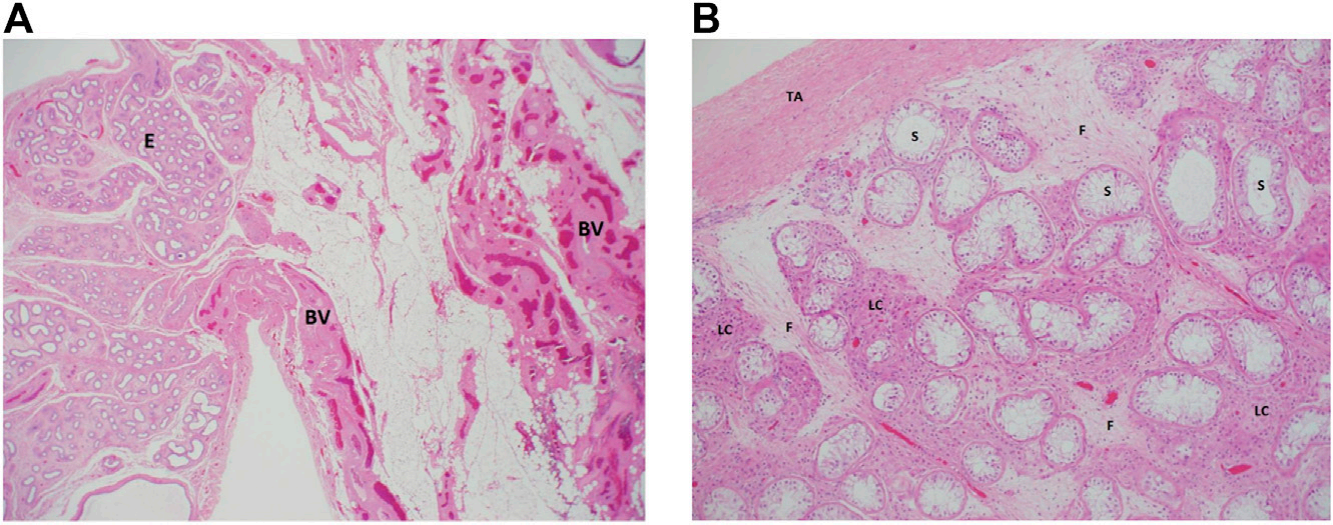
Histologic examination of the proband’s testicular biopsies. **(A)** Left testes: a low magnification (×20) from the surgical specimen of the left “testis” where epididymal tissue (E) is seen along with adjacent fatty tissue that is rich in blood vessels (BV). Note the total absence of any testicular tissue. **(B)** Right testis: an intermediate magnification (×100) image from the right testis showing seminiferous tubules (ST) that lack germ cells (lined by Sertoli cells only). The interstitium between the tubules shows clusters of hyperplastic Leydig cells (lLC) and fibrosis (F) secondary to tubular atrophy. TA, tunica albuginea. Both sections were stained using hematoxylin and eosin staining.

After extensive counseling of the patient and her parents, she underwent laparoscopy, left inguinal exploration, and bilateral orchiectomy (the left testis was found atrophied). The histopathological examination using hematoxylin and eosin staining showed a left undescended testis, atrophied with vasa efferentia, and epididymal tissue, negative for neoplasm at ×20 magnification (Figure 1A). The right testis showed total loss of germ cells in the seminiferous tubules, which were lined by mature Sertoli cells and negative for neoplasm or intratubular germ cell neoplasm at ×100 magnification (Figure 1B).

At age 15 years, the patient was started on hormonal replacement in the form of Premarin 0.625 mg days 1–25 of each month with medroxyprogesterone 10 mg daily days 16–25 of each month. With that, she started to have regular menstrual cycles and noticed further development of her breasts. Following orchidectomy, she also had regression of the clitromegly and significant breast development (Tanner IV). The patient is currently 21 years old. Her weight is 79 kg, height 162 cm, and BMI 30 kg/m^2^. She continues to feel well and is comfortable with her gender identity with excellent educational and social progress.

### Identification of Causal Mutation

**FIGURE 2:**
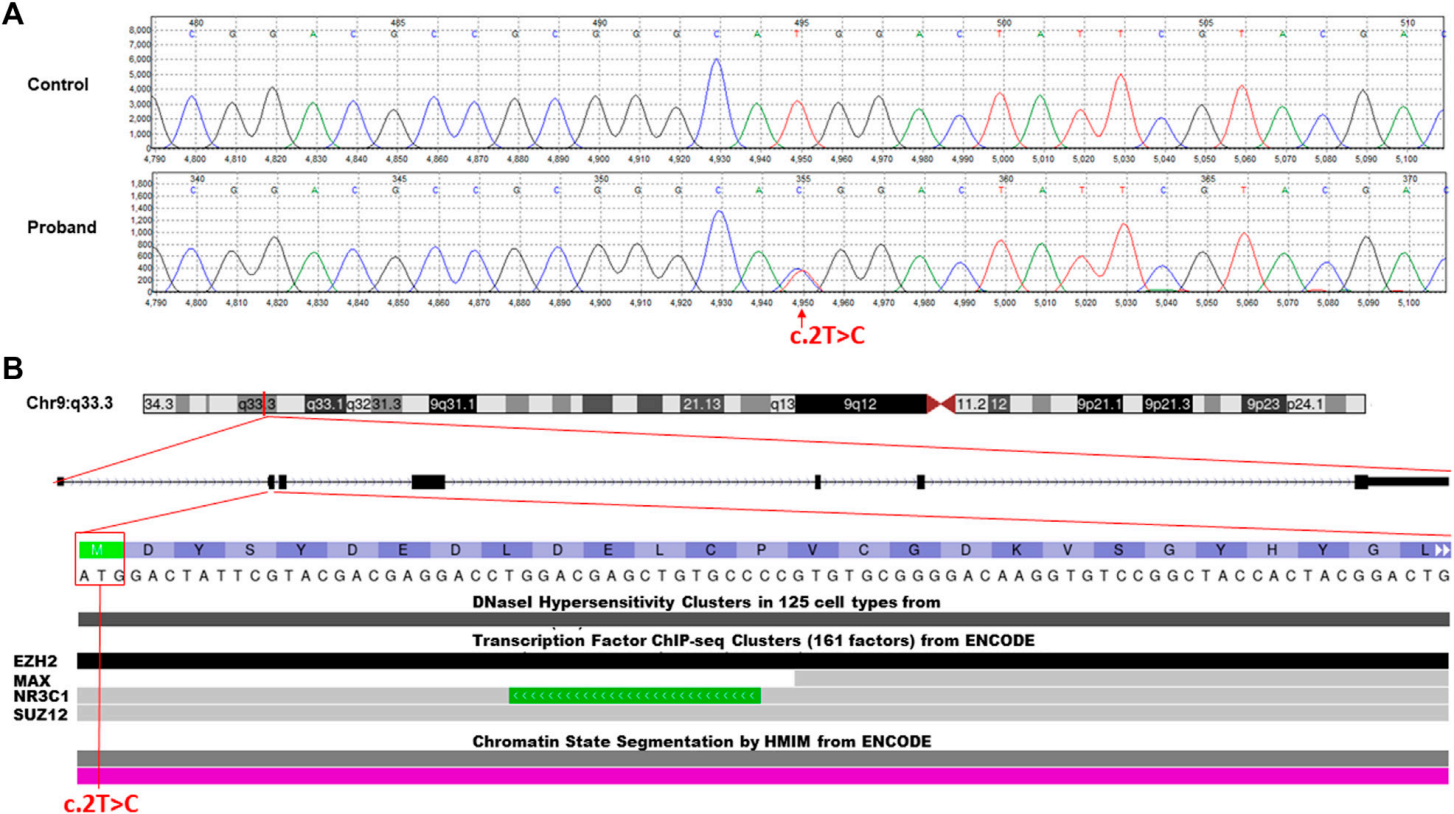
Molecular characterization of the identified c.2T>C missense variant in the *NR5A1* gene. **(A)** Chromatogram of Sanger sequencing segregation analysis of the wild-type control and affected proband. Red arrow represents the identified variant. **(B)** Annotation of the identified missense variant in the *NR5A1* gene (c.2T>C, p.Met1Thr) is located on chromosome 9p33.3. The variant falls in the first transcribed exon (exon 2) of the N-terminal region and interrupts the starting codon (ATG>ACG). The variant located in some of the ENCODE functional data tracks in the USCS genome browser (http://genome.ucsc.edu/). The variant interrupt DNaseI hypersensitivity and some predicted transcription factor binding sites (TFBSs) such as EZH2, NR3C1, and SUZ12.

The WES data analysis and Sanger sequencing (Figure 2A) revealed a heterozygous missense variant in the first transcribed exon of the *NR5A1* gene (NM_004959.5, c.2T>C, p.Met1Thr) This novel mutation was located on chr:127,265,673 and resulted in replacement of the first initiation codon ATG (methionine) by ACG (threonine).

### *In Silico* Classification of *NR5A1*-Identified Mutation

The classification analysis, including the pathogenicity analysis for this identified missense variant, was determined based on various *in silico* parameters to be pathogenic including the pathogenic scores obtained using SIFT (damaging); PolyPhen2, which was used to predict possible impacts of amino acid substitutions on protein structure and function (probably damaging); MutationTaster (disease causing); PROVEAN (damaging); and FATHMM (pathogenic). ENCODE annotation on the UCSC genome browser revealed that the variant falls in a functional region of *NR5A1* and thus predicted to be pathogenic (Figure 2B). The variant was predicted to fall in several transcriptional factor binding motifs such as EZH2, NR3C1, and SUZ12 (Figure 2B). The identified variant was not observed in the heterozygous state in the ExAC, 1000 Genomes, and genomAD and not in the Saudi Genome Project of >5,000 individual full exome sequencing data (https://shgp.kacst.edu.sa/index.en.html).

### Phylogenetic Conservation

**FIGURE 3:**
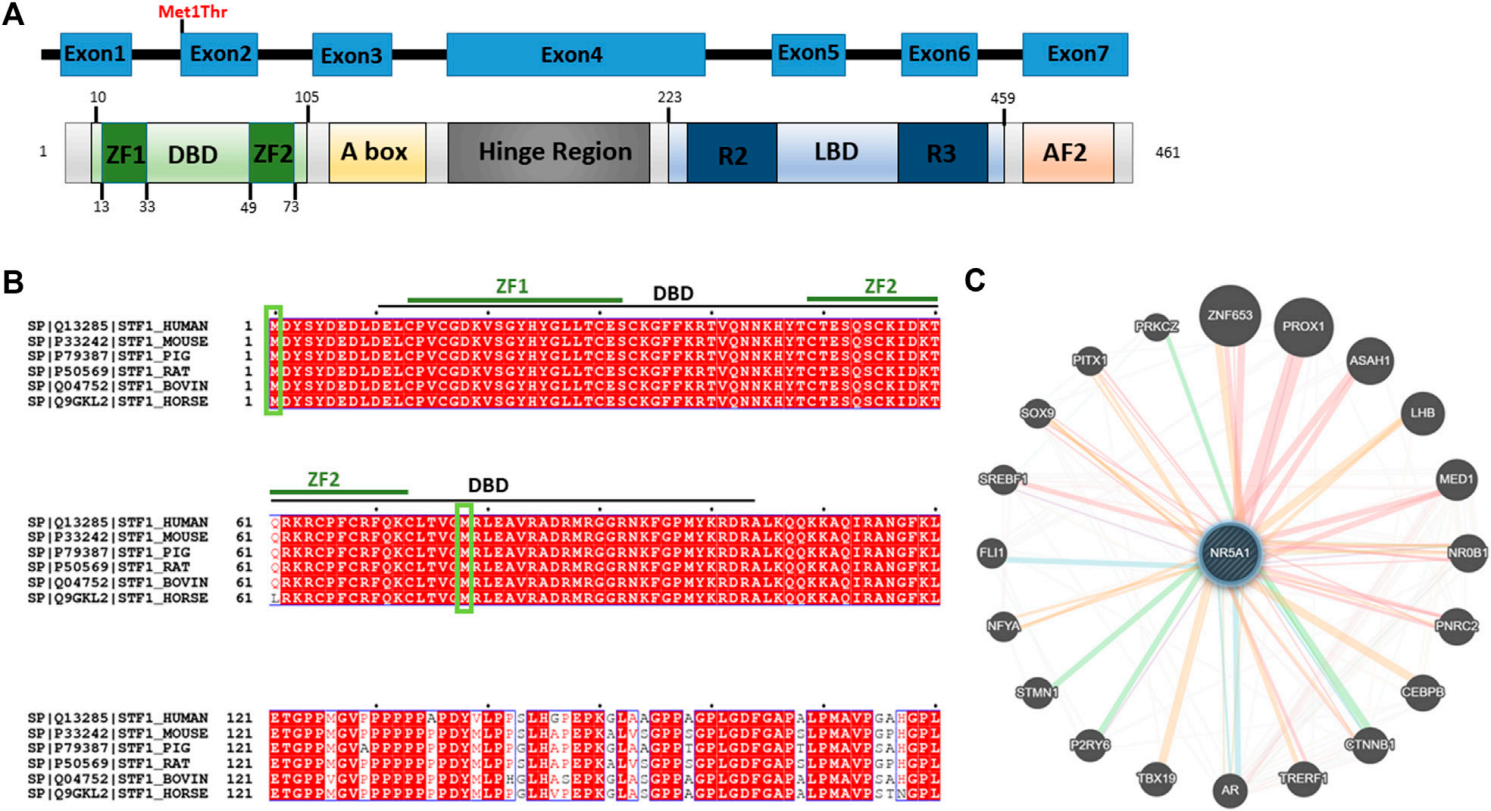
Predicted effect of Met1Thr mutation on NR5A1 function.** (A)** Schematic diagram of the identified variant at amino acid 1 is located on the DBD (green) in the N-terminus of NR5A1. The diagram was generated using PROSITE (https://prosite.expasy.org/cgi-bin/prosite). **(B)** Alignment of the sequences of H. Sapiens NR5A1 (UniProt: Q13285) with the Mouse NR5A1 (UniProt: P33242), Pig NR5A1 (UniProt: P79387), Rat NR5A1 (UniProt: P50569), Bovin NR5A1 (Uniprot: Q04752), and Horse NR5A1 (UniProt: Q9GKL2). The alignment was generated using UniProt Align (http://www.uniprot.org/align/). Start codon (Met) are represented by green rectangles. The alignment file was generated using UniProt Align (http://www.uniprot.org/align/), and final processing was performed in ESPript (http://espript.ibcp.fr). **(C)** Schematic representation of the NR5A1 interactions with other proteins using GeneMANIA (http://genemania.org/): physical interaction with other proteins (pink lines), genetics interaction (green lines), co-localization (blue lines), and predicted interaction (orange lines).

The variant falls in a functional region of the N-terminal region of *NR5A1* and disrupts the start codon in the first coding exon, exon 2 (Figure 3A). Full-length orthologous protein sequences from a range of animal species were retrieved from the UniProt database and aligned using UniProt Align, and final processing was performed in ESPript. Alignment of the sequence of H. Sapiens NR5A1 (UniProt: Q13285) with the Mouse NR5A1 (UniProt: P33242), Pig NR5A1 (UniProt: P79387), Rat NR5A1 (UniProt: P50569), Horse NR5A1 (UniProt: Q9GKL2) and Bovine NR5A1 (UniProt: Q04752) was generated (Figure 3B). The p.Met1Thr variant is located in a highly conserved position across mammals and higher eukaryotes, and thus, this strengthens the pathogenicity of the identified variant (Figure 3B). The STRING protein interaction shows that there is a strong interaction between NR5A1 and several other DSD proteins such as SOX9 (Figure 3C).

## Discussion

In this study, we describe an interesting case of 46,XY DSD. The patient has severe gonadal dysgenesis, and the likely pathogenesis is related to lack of androgens and anti-Müllerian hormone (AMH) from the severely dysgenetic testes during the first few weeks of life. Lack of androgens led to the development of the female external genitalia phenotype and probably was also the reason for the undescended testes. Lack of the AMH resulted in the formation of the uterus and upper third of the vagina. Although NR5A1 is an important factor for normal adrenocortical development, this patient had no evidence of adrenal insufficiency. Previous studies showed that *NR5A1* mutations, especially heterozygous ones, do not usually affect adrenocortical function, while homozygous mutations may be associated with gonadal and adrenocortical insufficiency (Lin et al., 2007; Song et al., 2018).

*NR5A1* is expressed in the bipotential gonads during the early gonadal development. It modulates the expression of critical genes for male gonadal development including *SRY* and *SOX9* (De Santa Barbara et al., 1998; Sekido and Lovell-Badge, 2008). It also activates the expression of the AMH that is responsible for regression of the Müllerian duct, the origin of internal female genitalia (Shen et al., 1994; Lasala et al., 2011). Finally, it stimulates testosterone biosynthesis by Leydig cells (Shen et al., 1994; De Santa Barbara et al., 1998). Therefore, impairment of its structure and function by mutations such as the one in our patient is likely to affect all of these regulatory functions and explains the clinical phenotype of our patient and similar cases. The clinical spectrum of patients with *NR5A1* mutations in subjects with a 46,XY karyotype is wide ranging from hypospadias only to a micropenis to a complete female external genitalia like that in our patient. This wide spectrum in the phenotypes of patients with *NR5A1* mutations is probably related to the type of mutations, their locations in the gene, and their effects on its function. In our patient, since the causative mutation involves the initiation codon, it probably has major effects on the gene function resulting in severe phenotype with complete sex reversal.

The novel *NR5A1* variant we detected in this patient is a heterozygous missense c.2T>C, (p.Met1Thr) variant located in the first transcribed exon (exon 2) of the N-terminal region and interrupts the starting codon (ATG>ACG). The disruption of the start codon in Met1Thr mutation affects the initial translation and formation of the DBD (10–105 aa) that contains two zinc finger motifs (ZF1: 13–33 aa; Zf2: 49–73 aa), but not the other domains in NR5A1, and thus may partially affect the overall function. Lourenço et al. (2009) have identified another variant that affects the initiation codon (c.3G>A, p.M1I) in two girls with 46,XY and 46,XX karyotypes. The first one (46,XY) was a 12-year-old girl who presented in a similar way to our patient with clitromegly, primary amenorrhea, and small breasts and uterus. Her surgically removed testes showed fibrous tissue, disorganized tubules, and Leydig cell hyperplasia. The second one (46,XX) was a 16-year-old girl who presented with secondary amenorrhea, small breasts, and no pubic hair, and her ovarian tissue showed only fibrous tissue with no follicles. Their 46-year-old mother reported normal menses. Genetic testing revealed an *NR5A1* mutation in the third nucleotide of the initiation codon (c.3G>A, p.M1I) in the two affected siblings and their mother but not in another normal sister and the father (Lourenço et al., 2009). The authors explained the absence of phenotypic abnormalities in the mother by the variable penetrance of the mutant allele due to variable translation defect, existence of modifier genes, or environmental defects as was previously seen in monozygotic twins with an *N5RA1* mutation and variable penetrance (Philibert et al., 2007). Similarly, in another report, Philibert et al. (2010) described a pathogenic heterozygous variant in the first nucleotide of the initiation codon (c.1A>G, p.M1V) in a female patient. It was speculated that this variant will abolish the transcriptional initiation and probably affects the protein activity.

Each domain of NR5A1 has been shown to play a critical and distinctive role. The DBD is crucial for transcription factor binding to promoters. The DBD contains a core with two Cys4 zinc finger motifs and a highly conserved Ftz-F1 box motif that is considered as a stabilizing region and involved in SF-1 to DNA interaction (Parker and Schimmer, 1997; Little et al., 2006). Owing to this impaired DBD, the protein–protein interaction and the anchoring will probably be affected, and the stabilizing ability might get disrupted. In addition, the impaired DBD might not have the ability to bind, interact, or trans-activate several proteins and subsequently could affect the overall NR5A1 nuclear receptor function. The STRING protein interaction revealed that NR5A1 physically interacts with SOX9. It has been demonstrated that NR5A1 trans-activates Sox9/SOX9 and nuclear receptor subfamily 0 group B member 1 (Nr0b1/NR0B1) through interacting with sex-determining region Y (SRY) (Sekido and Lovell-Badge, 2008). It has been demonstrated that during testicular development in mice, NR5A1 activates the testis enhancer sequence core element (TESCO) of Sox9/SOX9 (Sekido and Lovell-Badge, 2008). In addition, the impaired NR5A1 might lose the ability to modulate the genes that are involved in steroidogenesis such as steroidogenic acute regulatory protein (StAR) and cytochrome P450 steroid hydroxylase (CYP) enzymes including CYP11A1 and CYP17A1 in Leydig cells, which are required for testosterone biosynthesis (Lin and Achermann, 2008). In addition, NR5A1 was found to play a pivotal role in the transcriptional regulation of the expression of luteinizing hormone receptor (LHCGR) in both theca and granulosa cells (Luo et al., 1994). Furthermore, NR5A1 regulates cholesterol mobilization including AMH, HMGCoA synthase, and 3b-hydroxysteroid dehydrogenase (3bHSD). In contrast, it has been demonstrated that NR5A1 upregulates the insulin-like polypeptide 3 (INSL3) expression, which regulates testicular descent (Schimmer and White, 2010). However, owing to the presence of another start codon at position 78 (Figure 3B), the transcription of other NR5A1 domains such as A-box, LBD, AF-1, and AF-2 is probably unaffected. Therefore, this variant might not affect the transcriptional activation or suppression of some parts of the gene. It has been demonstrated that A-box binds to hormone response elements, which respond to steroid receptors and initiate the gene signaling pathway by the steroid hormones (Little et al., 2006). Therefore, this mutation may not cause hormonal alteration. It has been demonstrated that mutations located in the DBD (c.90T>G) and the DBD ZF1 box domain of the DBD (c.58G>C; c.70C>T and c.70delC) lead to affected individuals having partial or complete gonadal dysgenesis (Camats et al., 2012). Similar heterozygous c.18delC, V15M, C16X, C33S, and G35E variants located in the Ftz-ZF domain of the *NR5A*1 gene have also been documented in another study with a similar clinical phenotype (Köhler et al., 2008). Overall, it has been speculated that this variant may be subjected to partial or complete loss of a functional DBD in the NR5A1 protein.

This case adds to the rare and unique mutations of *NR5A1*. Although functional assessment was not performed, the *in silico* prediction was highly suggestive of the pathogenic nature of this variant. Since this variant involves the initiation codon, it is expected to have a detrimental effect on the protein translation. However, the final protein function pathogenicity effect may require further functional verification, which is not carried out in this study.

In conclusion, we have described the clinical and molecular genetics of a case of 46,XY DSD and identified a novel heterozygous missense c.2T>C variant changing the initiation codon of the *N5RA1* gene from methionine to threonine in this patient. This variant is predicted to impair the DBD and subsequently affect the NR5A1 nuclear receptor function.

## Data Availability

The datasets for this article are not publicly available due to concerns regarding participant/patient anonymity. Requests to access the datasets should be directed to the corresponding author.
